# Exploring functional dysconnectivity in schizophrenia: alterations in eigenvector centrality mapping and insights into related genes from transcriptional profiles

**DOI:** 10.1038/s41537-024-00457-1

**Published:** 2024-03-15

**Authors:** Yuan Ji, Mengjing Cai, Yujing Zhou, Juanwei Ma, Yijing Zhang, Zhihui Zhang, Jiaxuan Zhao, Ying Wang, Yurong Jiang, Ying Zhai, Jinglei Xu, Minghuan Lei, Qiang Xu, Huaigui Liu, Feng Liu

**Affiliations:** 1 Department of Radiology and Tianjin Key Laboratory of Functional Imaging, Tianjin Medical University General Hospital, Tianjin, China; 2 Department of Radiology, the First Affiliated Hospital of Dalian Medical University, Dalian, China

**Keywords:** Schizophrenia, Schizophrenia

## Abstract

Schizophrenia is a mental health disorder characterized by functional dysconnectivity. Eigenvector centrality mapping (ECM) has been employed to investigate alterations in functional connectivity in schizophrenia, yet the results lack consistency, and the genetic mechanisms underlying these changes remain unclear. In this study, whole-brain voxel-wise ECM analyses were conducted on resting-state functional magnetic resonance imaging data. A cohort of 91 patients with schizophrenia and 91 matched healthy controls were included during the discovery stage. Additionally, in the replication stage, 153 individuals with schizophrenia and 182 healthy individuals participated. Subsequently, a comprehensive analysis was performed using an independent transcriptional database derived from six postmortem healthy adult brains to explore potential genetic factors influencing the observed functional dysconnectivity, and to investigate the roles of identified genes in neural processes and pathways. The results revealed significant and reliable alterations in the ECM across multiple brain regions in schizophrenia. Specifically, there was a significant decrease in ECM in the bilateral superior and middle temporal gyrus, and an increase in the bilateral thalamus in both the discovery and replication stages. Furthermore, transcriptional analysis revealed 420 genes whose expression patterns were related to changes in ECM, and these genes were enriched mainly in biological processes associated with synaptic signaling and transmission. Together, this study enhances our knowledge of the neural processes and pathways involved in schizophrenia, shedding light on the genetic factors that may be linked to functional dysconnectivity in this disorder.

## Introduction

Schizophrenia has a heritability of up to 80%^1,2^, and commonly manifests as hallucinations, delusions, and cognitive impairments^3^. With a lifetime risk of approximately 1%, individuals with schizophrenia face an average reduction in lifespan of 14.5 years^4^. Abnormal functional connectivity in various brain regions has been observed in schizophrenia through the utilization of resting-state functional magnetic resonance imaging (rs-fMRI)^5–8^, indicating that schizophrenia is a pervasive dysconnectivity disorder^9–12^.

The traditional approaches for measuring resting-state functional connectivity include the seed-based approach and independent component analysis (ICA)^13^. In the seed-based approach, a specific brain region is chosen as the seed, and its time series correlation with other brain voxels or regions is examined^14^. While this method is useful for testing specific hypotheses, the selection of seed regions can be highly subjective^15^. Differing choices made by researchers in defining seeds pose challenges for making direct comparisons across studies^15,16^. On the other hand, ICA is a data-driven approach that identifies spatially independent components in functional neuroimaging data^17^, allowing the discovery of coherent patterns of brain activity without relying on predefined seed regions and serving as a valuable tool for uncovering intrinsic connectivity networks^18^. However, ICA primarily focuses on studying functional connectivity within brain networks, without specifically considering the information exchange between different networks. Therefore, there is an increasing need for an unbiased approach to assess alterations in brain functional connectivity in individuals with schizophrenia.

Eigenvector centrality mapping (ECM) has emerged as a promising and unbiased technique for thoroughly examining the functional connectivity throughout the entire brain^19,20^. Derived from graph theory, ECM computes the centrality of each node (such as a voxel or region) in the whole brain based on its connectivity pattern with the rest of the brain, without depending on predefined seed regions or networks. Previous studies have observed changes in ECM among individuals with schizophrenia. Wheeler et al. were the first to observe alterations in ECM, highlighting increased ECM in regions including the bilateral inferior frontal gyrus, supramarginal gyrus, superior temporal gyrus, as well as the right caudal middle frontal gyrus, superior frontal gyrus, superior parietal gyrus, and fusiform gyrus among patients with schizophrenia^21^. Subsequently, Skatun et al. reported decreases in ECM in the bilateral hippocampus, amygdala, putamen, occipital cortex, and somatosensory cortex, alongside increases in the bilateral frontal and parietal cortex^22^. The discrepancies observed in ECM studies investigating schizophrenia may arise from variations in node definition in brain networks^23^, and previous research has demonstrated the distinct advantages of modeling the brain network at the voxel level^24^. Furthermore, limited sample sizes and clinical heterogeneity represent additional sources of inconsistent findings, underscoring the need for independent datasets to validate these results.

Schizophrenia is recognized as a polygenic disorder, with an estimated heritability of approximately 80%^25,26^. Genome-wide association studies (GWAS) have revealed numerous genetic variants associated with schizophrenia, implicating genes involved in neurodevelopment, synaptic transmission, and immune system function as potentially playing critical roles in its pathogenesis^26,27^. In addition, both twin and GWAS studies have indicated that resting-state functional connectivity exhibits moderate to high heritability^28–30^. Consequently, it is imperative to elucidate the molecular mechanisms underlying ECM alterations in schizophrenia. Transcriptome analysis of the human brain is an emerging field dedicated to exploring the transcriptional landscape of genes within the human brain^31–34^. Through a systematic examination of gene expression, transcriptome analysis aims to unveil variations in gene activity under different disease conditions, offering crucial insights into the understanding of brain structure and functional alterations^35–38^.

In the present study, our aim was to investigate reliable and reproducible whole-brain voxel-wise ECM alterations in schizophrenia and explore the underlying genetic determinants. During the discovery stage, 91 patients with schizophrenia and 91 matched healthy controls were recruited for the analysis of ECM changes. For the replication stage, a public dataset comprising 153 individuals with schizophrenia and 182 healthy individuals was used to validate the ECM alterations identified in the discovery stage. This two-stage approach not only strengthens the reliability of the observed changes but also enables a more extensive evaluation of their consistency across diverse datasets. Subsequently, leveraging the Allen Human Brain Atlas^39^ (AHBA, http://human.brain-map.org) database, which contains a densely sampled collection of postmortem human brain tissue, gene expression across various brain regions was analyzed through transcriptome analysis to uncover the molecular mechanism of ECM changes in schizophrenia. The dual focus on both ECM alterations and genetic associations contributes to a comprehensive understanding of the complex etiology of schizophrenia.

## Materials and methods

### Participants and MRI data acquisition

#### Discovery stage

We employed case-control datasets sourced from Tianjin Medical University General Hospital (TMUGH) as our discovery dataset. This study received approval from the local Ethics Committee, and each participant provided informed written consent. Initially, we included data from 95 individuals diagnosed with schizophrenia based on the criteria of the Structured Clinical Interview for Diagnostic and Statistical Manual of Mental Disorders Fourth Edition (SCID-DSM-IV) and 93 healthy individuals without a history of neurological or psychiatric disorders, as well as the absence of any gross abnormalities. The exclusion criteria of all subjects included: (1) left-handedness; (2) aged less than 18 years or more than 60 years; (3) MRI contraindications; (4) a history of central nervous system disorders (e.g., epilepsy), systemic illnesses (e.g., cardiovascular disease, diabetes mellitus), or substance (e.g., hypnotics, alcohol) abuse; and (5) the presence of intracranial organic lesions. Moreover, we excluded healthy controls whose first-degree relatives had a history of any mental disorders.

MRI data were acquired by a 3.0-T MR system (Discovery MR750, General Electric, Milwaukee, WI, USA) at TMUGH. To minimize head movement and scanner noise, tight but comfortable foam padding and earplugs were utilized. Sagittal 3D T1-weighted images were acquired by a brain volume sequence with the following parameters: repetition time (TR) = 8.2 ms; echo time (TE) = 3.2 ms; inversion time (TI) = 450 ms; flip angle (FA) = 12°; field of view (FOV) = 256 mm × 256 mm; matrix = 256 × 256; slice thickness = 1 mm; no gap; and 188 sagittal slices. The rs-fMRI data were acquired using a single-shot echo planar imaging (SS-EPI) sequence with the following parameters: TR = 2000 ms; TE = 30 ms; FA = 90°; FOV = 256 × 256 mm; matrix = 128 × 128; slice thickness = 3 mm; gap = 1 mm; 32 slices; and 180 volumes.

#### Replication stage

We downloaded a public dataset from the SchizConnect database (http://schizconnect.org/) for replication^40^, which included the BrainGluSchi^41^, COBRE^41^, and NMorphCH projects^42^. The details of participant inclusion/exclusion criteria and MRI acquisition parameter are listed as follows.

In the BrainGluSchi dataset, there were 71 individuals with schizophrenia and 80 healthy individuals available. The inclusion criteria included: (1) Diagnosis of schizophrenia determined through consensus by two research psychiatrists using the SCID-DSM-IV; (2) if receiving treatment, being clinically stable on the same antipsychotic medications for > 4 weeks. Exclusion criteria included: (1) confirmed or suspected pregnancy; (2) a history of central nervous system disorders; (3) mental deficiency, (4) current substance use disorder (except nicotine). T1-weight MRI data were acquired using a 3.0-T Siemens scanner with the following parameters: TR = 2530 ms; TE = 1.64 ms; TI = 1200 ms; FA = 7°; FOV = 256 mm × 256 mm; matrix = 256 × 256; slice thickness = 1 mm. The parameters for rs-fMRI acquisition with 3.0-T Siemens scanner were listed as follows: TR = 2000 ms; TE = 29 ms; FA = 75°; FOV = 240 mm × 240 mm; matrix = 64 × 64; slice thickness = 3.5 mm.

The COBRE dataset included 80 individuals with schizophrenia and 84 healthy individuals. Patient inclusion criteria involved a diagnosis of schizophrenia or schizoaffective disorder according to SCID-DSM-IV, between the ages of 18 and 65. Exclusion criteria included: (1) a history of neurological disorder, (2) a history of mental retardation, (3) a history of severe head trauma with more than 5 min loss of consciousness, (4) a history of substance abuse or dependence within the last 12 months. T1-weight MRI data were acquired using a 3.0-T Siemens scanner with the following parameters: TR = 2530 ms; TE = 1.64 ms; TI = 1200 ms; FA = 7°; FOV = 256 mm × 256 mm; matrix = 256 × 256; slice thickness = 1 mm. The parameters for rs-fMRI acquisition with 3.0-T Siemens scanner were listed as follows: TR = 2000 ms; TE = 29 ms; FA = 75°; FOV = 240 mm × 240 mm; matrix = 64 × 64; slice thickness = 3.5 mm.

The NMorphCH dataset consisted of 41 individuals with schizophrenia and 38 healthy individuals. Patients were diagnosed by SCID-DSM-IV. Structural MRI data were acquired using a 3.0-T Siemens scanner with the following parameters: TR = 3.15 ms; TE = 1.37 ms; FA = 8°; FOV = 256 × 256 mm; matrix = 162 × 162; slice thickness = 1.6 mm; 188 sagittal slices. The parameters for rs-fMRI acquisition with 3.0-T Siemens scanner were listed as follows: TR = 2200 ms; TE = 27 ms; FA = 90°; FOV = 100 mm × 100 mm; matrix = 384 × 384; slice thickness = 4 mm.

### MRI data preprocessing

Two researchers independently performed quality control checks on the MRI data, eliminating data that lacked coverage over the entire spatial extent of brain tissue, as well as instances with head motion and aliasing artifacts, through visual inspection. The rs-fMRI data were preprocessed via the following procedures. We first excluded the initial 10 volumes to ensure signal equilibrium, followed by correction for temporal differences between slices and head motion. Participants with excessive head movements (maximum translation >2.0 mm or rotation >2.0 degrees in any direction) were excluded from the subsequent analyses. In addition, the mean Jenkinson framewise displacement (FD)^43^ was calculated, and subjects with a mean FD > 0.5 were discarded. To further remove motion-related artifacts from fMRI data, we employed independent component analysis-based automatic removal of motion artifacts (ICA-AROMA)^44^, which demonstrated cost-effectiveness across various benchmarks compared to censoring-based methods^45^. Following this, individual structural images were co-registered to the mean functional image, and the transformed structural images were segmented into gray matter, white matter, and cerebrospinal fluid. Utilizing these segmented images, the diffeomorphic anatomical registration through exponentiated lie algebra (DARTEL) tool^46^ was employed to estimate normalization parameters, facilitating the transformation from individual native space to the Montreal Neurological Institute (MNI) space. Nuisance covariates, encompassing linear trends, white matter signals, and cerebrospinal fluid signals, were then regressed out. Finally, temporal band-pass filtering (0.01–0.1 Hz) was applied to mitigate the effects of low-frequency drift and high-frequency noise, followed by normalization to MNI space using the aforementioned parameters and resampling to 3-mm isotropic voxels.

### ECM calculation

The fast ECM software (https://github.com/amwink/bias/tree/master/matlab/fastECM) was employed to compute whole-brain voxel-wise ECM. Traditional ECM methods necessitate the computation of a voxel-wise connectivity matrix to derive its eigenvector^19^. However, the fast ECM method performs matrix-vector products directly without the need to compute or store the connectivity matrix, utilizing an efficient power iteration algorithm to detect the dominant eigenvector^47^. Moreover, it eliminates the need for thresholding or binarizing connectivity matrices, ensuring uniform network topology and size across all subjects. Following ECM computation, each participant obtained a voxel-wise ECM map. Note that ECM computation was confined within a gray matter mask, which includes approximately 66,000 voxels covering the entire brain.

### Case-control ECM alterations

Given that the replication dataset was sourced from three distinct centers (BrainGluSchi, COBRE, and NMorphCH), we applied the Combat approach (https://github.com/Jfortin1/ComBatHarmonization) to harmonize the data^48^. The harmonization process, employed to mitigate variations from different scanners or scanning parameters, has the capability to enhance statistical power, retrieve accurate effect sizes, and demonstrate robustness^48^. All the ECM maps from discovery and replication after harmonization were smoothed with a Gaussian kernel of 8 × 8 × 8 mm full-width at half maximum.

Group-level comparisons were independently conducted in the discovery and replication stages. Specifically, between-group ECM comparisons were conducted for each dataset using the Data Processing & Analysis for Brain Imaging (DPABI) toolbox^49^, controlling for age, sex, and mean FD. The statistical analysis employed a subject-based nonparametric permutation test with 1000 permutations and incorporated the threshold-free cluster enhancement (TFCE) method^50^ to control the family-wise error (FWE) rate, and the significance level was set at TFCE-FWE *P* < 0.05. Regions exhibiting significant ECM changes in the same direction in both the discovery and replication stages were considered reliable ECM alterations.

### Gene expression data processing

The standardized microarray expression data with 3702 tissue samples, derived from six postmortem adult brains without any known neuropsychiatric or neuropathological history, were downloaded from the AHBA database^39^. The demographic details for each donor are presented in Table S1. Since tissue samples from the right hemisphere were collected from only two donors, while samples from the left hemisphere were obtained from six donors, only left hemisphere samples were included for preprocessing^51^. This was accomplished using abagen^52^ (https://www.github.com/netneurolab/abagen), an open-source toolbox that provides consistent workflows for processing and formatting gene expression data based on established recommendations. First, microarray probes were reannotated to genes based on the annotation information provided by ref. ^53^. Second, probes with expression levels falling below the background signal across more than 50% of the tissue samples were eliminated. Third, the probe demonstrating the highest differential stability (i.e., displaying the most consistent regional expression pattern across the six donated brains) was chosen to represent each gene. Fourth, the microarray tissue samples were matched to anatomical regions within the gray matter mask based on MNI coordinates. Fifth, normalization of expression values was carried out by employing a scaled robust sigmoid function to standardize expression values for each tissue sample and donor across genes, as well as for each gene and donor across tissue samples. Finally, separate normalization procedures for cortical and subcortical structures were conducted to address variations in microarray expression across major structural compartments (e.g., cortex vs. subcortex)^53,54^. These processing protocols resulted in an expression matrix of dimensions 15,633 genes by 2296 tissue samples.

### Identifying genes related to ECM alterations in schizophrenia

Transcriptome analysis was conducted to explore the genetic mechanisms underlying ECM changes in schizophrenia. First, the tissue samples were categorized into two groups: regions with reliable ECM alterations (identified in both the discovery and replication stages) and regions without such alterations. The Mann–Whitney *U* test was subsequently used for each gene to compare expression values between tissue samples from regions with and without reliable ECM alterations. A gene was considered to have a significant difference in expression levels if *P* < 0.05/15,633 (corrected for the total number of genes) using Bonferroni correction.

### Functional annotation

Functional annotation analyses were performed to investigate the biological functions and pathways for the identified genes. First, Toppgene^55^ (https://toppgene.cchmc.org/) was used to identify significant enrichment in gene ontology (GO). Until now, the Toppgene website database provides 20,649 background genes for biological process enrichment, 20,915 background genes for cellular component enrichment, and 19,912 background genes for molecular function enrichment in GO analysis (https://toppgene.cchmc.org/navigation/database.jsp). To account for multiple comparisons, the Benjamini and Hochberg method for false discovery rate (FDR) was applied with a significance threshold of *P* < 0.05. Second, to explore the protein functionalities downstream of genes with higher and lower expression in the brain regions exhibiting significant ECM alterations in individuals with schizophrenia compared to regions without such alterations, we separately created protein-protein interaction (PPI) networks for these two gene sets using STRING^56^ version 12.0 (https://string-db.org/) with a confidence value of 0.9. Finally, the ChIP-X enrichment analysis tool ChEA3^57^ (https://maayanlab.cloud/chea3/) was employed to separately identify transcription factor (TF) and TF-TF co-regulatory networks for gene sets with higher and lower expression based on GTEx library. A TF with an odds ratio (OR) exceeding one indicates its role as an activator, while an OR less than one suggests the TF acts as a repressor^57^. Wilcoxon rank-sum tests were performed on the ORs of TFs to investigate differences between genes with increased or decreased expression.

## Results

### Participant characteristics

After quality control procedures, for the discovery stage, we included 91 individuals with schizophrenia and 91 healthy individuals from the TMUGH cohort; as for the replication stage (totally 153 patients and 182 controls), we included the BrainGluSchi project (57 individuals with schizophrenia and 75 healthy individuals), the COBRE project (64 individuals with schizophrenia and 76 healthy individuals), and the NMorphCH project (32 individuals with schizophrenia and 31 healthy individuals). The detailed demographic information for both patients and controls are shown in Table S2.

### ECM alterations in schizophrenia

**Fig. 1:**
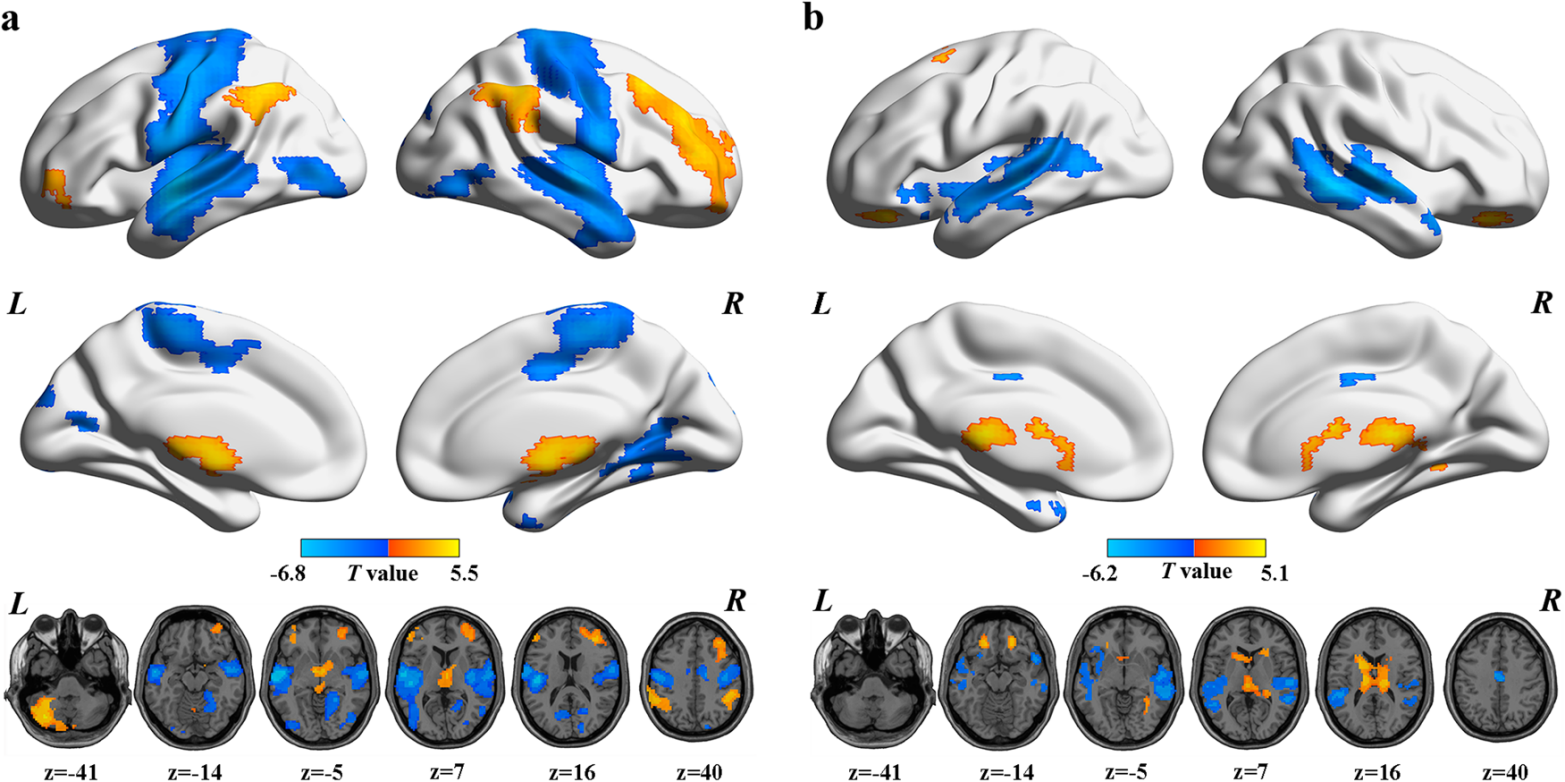
ECM differences between schizophrenia and healthycontrols. Brain regions exhibiting significant alterations in the ECM in schizophrenia were identified (*P* < 0.05, TFCE-FWE corrected) in the **a** discovery and **b** replication stages. The color bar corresponds to *t*-values, where a positive *t*-value represents increased ECM in schizophrenia than in healthy controls, and a negative *t*-value represents decreased ECM in schizophrenia than in healthy controls. ECM eigenvector centrality mapping, FWE family-wise error, L left, R right, TFCE threshold-free cluster enhancement.

In both the discovery and replication datasets, we compared voxel-wise differences in ECM within gray matter regions between individuals with schizophrenia and healthy controls. In the discovery stage, individuals with schizophrenia exhibited reduced ECM (one-sided *P* < 0.05, TFCE-FWE corrected) in various brain regions, including the bilateral postcentral gyrus, precentral gyrus, superior and middle temporal gyrus, insula, inferior occipital cortex, calcarine cortex, and left middle occipital cortex, as well as in the right temporal pole, lingual cortex, superior occipital gyrus, and cerebellum anterior lobe (Fig. 1a and Table S3). Conversely, individuals with schizophrenia showed increased ECM (one-sided *P* < 0.05, TFCE-FWE corrected) in the bilateral cerebellum Crus1 and Crus2, thalamus, middle frontal cortex, inferior parietal cortex, left inferior frontal gyrus, and the right superior frontal cortex (Fig. 1a and Table S3).

In the replication stage, individuals with schizophrenia showed decreased ECM (one-sided *P* < 0.05, TFCE-FWE corrected) in the bilateral superior and middle temporal gyrus, insula, hippocampus, median cingulate, paracingulate gyri, and the temporal pole (Fig. 1b and Table S4). In contrast, individuals with schizophrenia demonstrated an increased ECM (one-sided *P* < 0.05, TFCE-FWE corrected) in the bilateral orbitofrontal cortex, superior and middle frontal cortex, thalamus, and caudate (Fig. 1b and Table S4). The reliable ECM alterations observed in both the discovery and replication stages included reduced ECM in the bilateral superior and middle temporal gyrus, alongside increased ECM in the bilateral thalamus among individuals with schizophrenia.

### Genes related to ECM alterations in schizophrenia

**Fig. 2:**
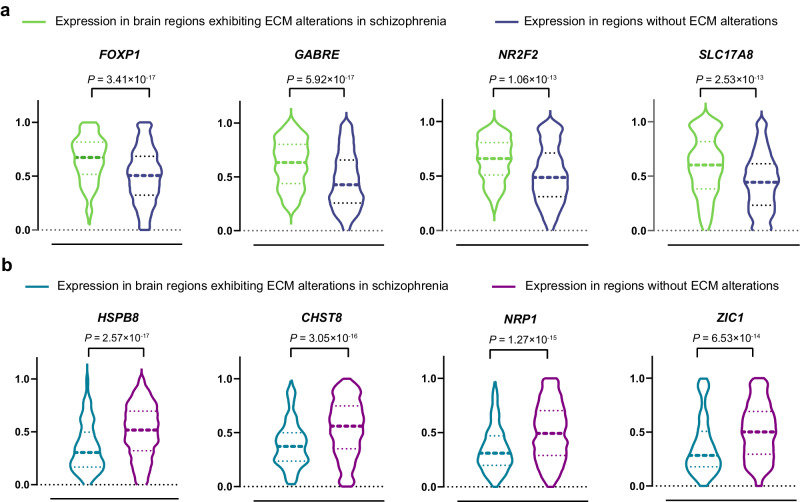
There were significant differences in the expression levels of eight representative genes between brain regions exhibiting ECM alterations in schizophrenia and regions without such alterations. Genes with (**a**) positive *z*-statistics and (**b**) negative *z*-statistics show increased and decreased expression in ECM-altered regions in individuals with schizophrenia, respectively. The *x*-axis in each violin plot distinguishes between two groups: ECM-altered regions in schizophrenia (left side) and other gray matter brain regions (right side). The *y*-axis shows the expression values for each tissue sample in the respective groups. At the top of each violin plot, the names of representative genes and the two-sided *P* values of Mann–Whitney *U* tests are displayed. The dashed line in each violin plot represents the median expression level, and the dotted lines represent the upper and lower quartiles. ECM eigenvector centrality mapping.

Among 2296 tissue samples, 194 tissue samples were located in gray matter regions exhibiting ECM alterations in schizophrenia, while the remaining 2102 tissue samples were located in gray matter regions without such ECM alterations. A total of 420 genes with significantly different expression levels (two-sided *P* < 0.05/15,633 = 3.20 × 10^−6^) were identified in brain regions exhibiting ECM alterations in schizophrenia, compared to regions without such ECM alterations. Among these genes, 116 had positive *z*-statistics (Table S5), indicating that their expression within the ECM-altered regions in schizophrenia was greater than regions without ECM alterations (Fig. 2a). For instance, the expression level of *FOXP1* was significantly greater (two-sided *P* = 3.41 × 10^−17^) in brain regions exhibiting ECM alterations in schizophrenia. *FOXP1* plays a crucial role in coordinating signaling pathways related to neurogenesis and neurodevelopmental disorders, thereby impacting the generation of excitatory cortical neurons, especially in basal radial glial cells^58^. Conversely, 304 genes had negative *z*-statistics (Table S6), suggesting that the expression levels of these genes within the ECM-altered regions in schizophrenia were lower than regions without such ECM alterations (Fig. 2b). As an example, *CHST8* showed a decreased expression level (two-sided *P* = 3.05 × 10^−16^) in ECM-altered regions in schizophrenia and has been implicated in potentially mediating the effects of antipsychotic treatments in schizophrenia, particularly in the domain of working memory^59^.

### Enrichment analysis

**Fig. 3:**
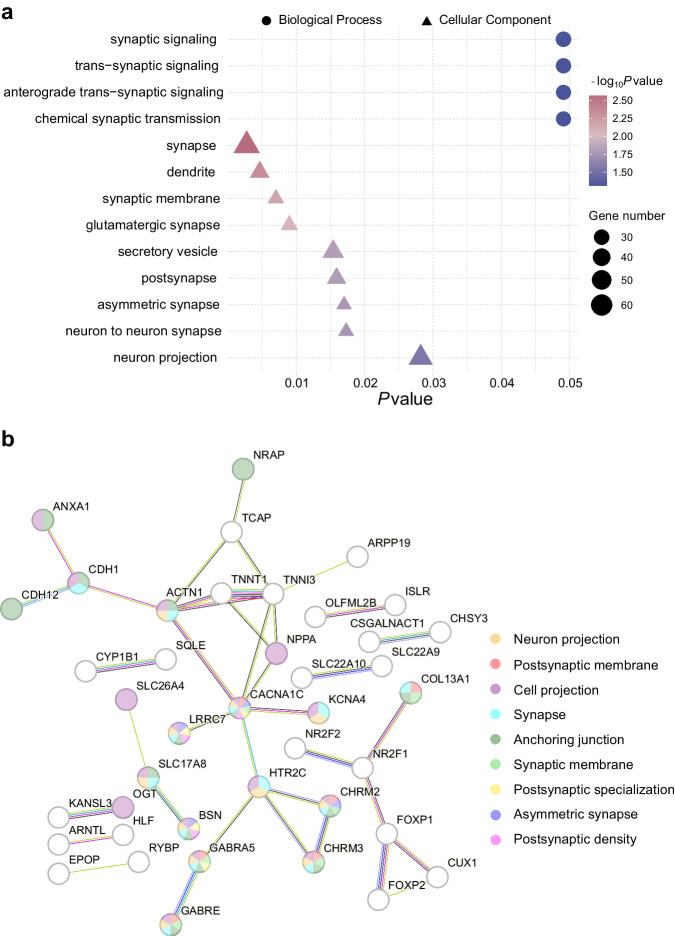
Enrichment analysis of the genes related to ECM alterations in patients with schizophrenia. **a** The bubble plot displays the enriched GO terms (one-sided *P* < 0.05, FDR corrected) of biological processes (circle) and cellular components (triangle) for genes related to ECM alterations in schizophrenia. The *x*-axis indicates the significance of each GO term (*y*-axis). The bubble size corresponds to the number of genes enriched for each term, and the color corresponds to the significance of each term in the statistical over-representation analysis. **b** The PPI network consisting of 41 proteins (depicted as spheres) and 38 edges was constructed using 116 genes with higher expression levels in brain regions exhibiting ECM alterations in schizophrenia compared to other gray matter regions. The network is centered around *CACNA1C*, which is enriched in multiple cellular components represented by different colors. ECM eigenvector centrality mapping, FDR false discovery rate, GO Gene Ontology, PPI protein-protein interaction.

The 420 altered ECM-related genes exhibited significant enrichment in biological processes encompassing synaptic signaling, trans-synaptic signaling, anterograde trans-synaptic signaling, and chemical synaptic transmission. In addition, there was significant enrichment in cellular components, including synapse and glutamatergic synapse, neuron projection, as well as dendrites, highlighting the biological significance of these processes (Fig. 3a, Table S7).

PPI networks were constructed for genes that exhibited differential expression higher and lower, respectively, in regions with significant ECM alterations compared to regions without such alterations. Specifically, the 116 genes with higher expression contributed to the formation of a PPI network comprising 41 nodes and 38 edges, which significantly exceeded the expected 23 edges (one-sided *P* = 3.30 × 10^−3^) (Fig. 3b). This network was centered around *CACNA1C* and was significantly enriched in the cell components of cell projection, synapse, anchoring junction, neuron projection, and postsynaptic membrane. The remaining 304 genes with lower expression resulted in the formation of a PPI network with 164 nodes and 248 edges, significantly exceeding the expected 177 edges (one-sided *P* = 2.29 × 10^−7^) (Supplementary Fig. 1). *FH* was one of the central nodes in this network, and the network was enriched in cellular components of mitochondria and the mitochondrial matrix.

**Fig. 4:**
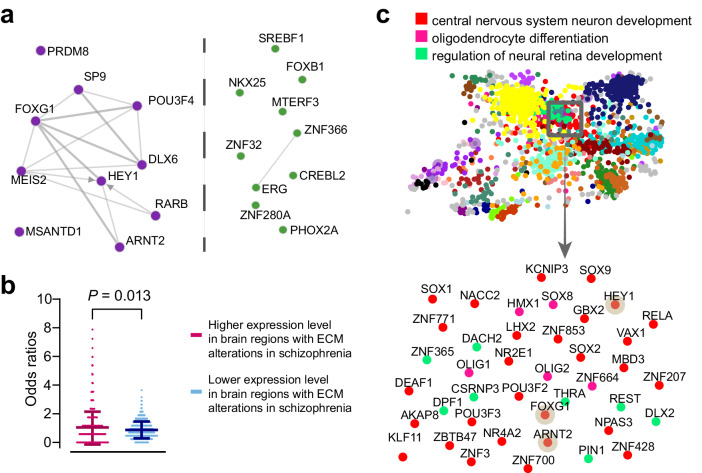
Prioritization of TFs based on genes with higher expression levels in brain regions exhibiting ECM alterations in schizophrenia compared to other gray matter regions. **a** TF-TF co-regulatory networks, with each node representing a TF and edges indicating communication between TFs to reflect their co-regulation, were generated from the top ten GTEx expression results. The left panel represents TFs predicted from 116 genes with higher expression in ECM-altered brain regions in schizophrenia, while the right panel represents TFs predicted from 304 genes with lower expression. **b** The significant difference in ORs (*y*-axis) for 1607 TFs is shown with the top *P* value from the Wilcoxon rank-sum test. The left part (magenta) represents TFs predicted by 116 genes with higher expression in ECM-altered brain regions in schizophrenia, and the right part (blue) represents TFs predicted by 304 genes with lower expression. **c** A global edgeless TF co-expression network (upper panel) plot from the GTEx database colored according to GO enrichment. The contents within the gray solid line box are magnified and displayed in the lower panel, including three of the top ten ranked TFs (nodes with brown shading), which are enriched in the development of neurons in the central nervous system. ECM eigenvector centrality mapping, GO Gene Ontology, ORs odds ratios, TF transcription factor.

TFs were also identified separately for the gene sets with higher and lower expression. Notably, genes with higher expression reveal a more enriched co-regulatory network of TFs, whereas those with lower expression show a less enriched co-regulatory network (Fig. 4a). The network, constructed based on the TFs predicted by genes with higher expression in ECM-altered regions in schizophrenia, is centered around *HEY1* and *DLX6*, both of which are associated with neurogenesis and forebrain and craniofacial development^60,61^. Significant differences in the ORs of these predicted TFs were further observed (Wilcoxon rank sum test: two-sided *P* = 0.013) (Fig. 4b, Tables S8, S9), indicating the presence of more activators in genes with greater expression levels in ECM alteration regions compared to regions without ECM alterations. Additionally, the TF co-expression networks could offer the top-ranking TFs in the broader human transcriptional GTEx regulatory network, and an observation revealed the enrichment of *HEY1*, *FOXG1*, and *ARNT2* in the model of central nervous system neuron development (Fig. 4c).

## Discussion

In this study, we investigated ECM alterations in schizophrenia and explored the underlying genetic determinants. To ensure the robustness of our results across different datasets, our analytic framework consisted of two main stages: an initial discovery stage, which included 91 patients and 91 controls, followed by a replication stage with 153 patients and 182 controls. Specifically, we observed a reproducible decrease in ECM in the bilateral superior and middle temporal gyrus, while an increase was detected in the bilateral thalamus in individuals with schizophrenia. Furthermore, our transcriptome analysis identified significant genes, including *FOXP1* and *CHST8*, providing insights into the molecular mechanisms underlying ECM changes in schizophrenia.

The bilateral superior and middle temporal gyrus, recognized for their pivotal roles in various cognitive processes including auditory processing^62^, language comprehension^63^, and social cognition^64^, have consistently been implicated in schizophrenia, associated with auditory hallucinations^65^, language disturbances^66^, and deficits in social functioning^67^. The observed decreases in ECM suggest disruptions in connectivity patterns within these regions, potentially contributing to the cognitive and perceptual abnormalities characteristic of schizophrenia. Conversely, the bilateral thalamus, serving as a key relay station in the brain, relaying sensory and motor signals to the cortex and regulating consciousness and alertness^68–70^, has also been implicated in the pathophysiology of schizophrenia^71^, contributing to sensory processing abnormalities and cognitive deficits^72,73^. The observed increases in ECM within the thalamus may reflect aberrant hyperconnectivity or compensatory mechanisms in response to disrupted cortical-thalamic circuits in schizophrenia. These findings emphasize the complex nature of functional dysconnectivity in schizophrenia, involving both hypoconnectivity and hyperconnectivity patterns across different brain regions^74^. Furthermore, the reproducibility of these ECM alterations across multiple datasets strengthens the reliability of our findings and underscores the robustness of these connectivity abnormalities in individuals with schizophrenia. Collectively, these findings suggest that targeting these affected brain regions in therapy may help to restore or modulate their connectivity, potentially leading to improvements in cognitive and perceptual functions in individuals with schizophrenia.

The transcriptional analysis unveiled genetic underpinnings associated with ECM alterations in schizophrenia, supporting the neurotransmitter hypothesis of the disorder. For example, *SLC17A8*, responsible for transporting the neurotransmitter glutamate into synaptic vesicles prior to its release into the synaptic cleft^75^, has demonstrated heightened expression levels in regions exhibiting altered ECM in schizophrenia. The association between schizophrenia and the glutamatergic neurotransmission pathway has been validated by extensive research^76,77^, and an elevation in the levels of the glutamate metabolite glutamine in the thalamus has also been noted^78^. Additionally, our findings highlight the key role of the *HEY1* in the TF co-regulatory network. Dopamine dysfunction, long implicated in schizophrenia’s pathophysiology^76,77^, was further supported by studies showing up-regulation of dopamine transporters with a VNTR domain and altered spontaneous activity upon *HEY1* knockout in mice^79^. Moreover, *CACNA1C* emerged as the hub gene in the PPI network formed from genes with higher expression in brain regions with ECM alterations in schizophrenia. Large-scale GWASs have identified *CACNA1C* as a risk gene for multiple psychiatric disorders, including schizophrenia^80–82^. By elucidating the molecular mechanisms underlying schizophrenia, further efforts can be directed towards developing targeted therapeutic strategies customized to address the specific molecular abnormalities observed in affected individuals.

Several limitations should be noted in this study. First, the transcriptional and neuroimaging data were not obtained from the same individuals, potentially introducing discrepancies due to inter-individual variability, thus impacting the robustness and generalizability of our findings. Nevertheless, certain genes demonstrate consistent spatial expression patterns across various brain structures among different individuals^54^. Additionally, numerous studies have demonstrated connections between transcriptional profiles and neuroimaging data in separate individuals^83–85^. Hence, this approach emerged as the most feasible method for us to establish correlations between gene expression profiles and neuroimaging data. Second, the current study cannot establish causal associations between neuroimaging alterations and gene expression, highlighting the need for further experimental validation to elucidate the underlying mechanisms driving these observed correlations. Third, the cross-sectional design limits our ability to track changes over time within individuals, potentially overlooking individual variability and the progression of schizophrenia-related dysconnectivity. Longitudinal studies would be essential to address these limitations and provide a more comprehensive understanding of the dynamics of schizophrenia-related dysconnectivity in the future. Finally, the inconsistencies in previous studies regarding ECM findings between schizophrenia and healthy controls may also be attributed to other factors such as varying MRI scanner and its parameters, as well as different data preprocessing pipelines. Future studies should aim to collect homogeneous samples, standardize scanning machines and parameters, and employ consistent analytical methods to address these inconsistencies.

## Conclusion

In conclusion, this study provided a reliable neuroimaging analysis of ECM alterations in schizophrenia, identifying 420 genes with distinct expression patterns associated with robust changes in the ECM. These genes were enriched in synaptic signaling and transmission processes, shedding light on the complex interplay between brain connectivity and genetic factors in schizophrenia. Overall, this study enhances the understanding of the complex mechanisms involved in the etiology of schizophrenia.

### Supplementary information

Supplementary Figure

Supplementary Tables

## Data Availability

The replication dataset was available from the SchizConnect database (http://schizconnect.org/). The corresponding author can provide access to the discovery dataset analyzed during the current study upon reasonable request.

## Code availability

All the software and code used in this study are publicly available. The structural MRI process and amygdala and subnucleus segmentation were performed using SPM12 (https://www.fil.ion.ucl.ac.uk/spm/software/spm12/) and ICA-AROMA (https://github.com/maartenmennes/ICA-AROMA). The harmonization was conducted using Combat harmonization (https://github.com/Jfortin1/ComBatHarmonization). The ECM were calculated by the fast ECM (https://github.com/amwink/bias/tree/master/matlab/fastECM). The preprocessing for the gene expression data was conducted by Abagen (https://www.github.com/netneurolab/abagen). The functional annotation involved in ToppGene (https://toppgene.cchmc.org/), STRING (https://string-db.org/) and ChEA3 (https://maayanlab.cloud/chea3/).

## References

[1] Chen J (2019). Shared genetic risk of schizophrenia and gray matter reduction in 6p22.1. Schizophr. Bull..

[2] Mowry BJ, Gratten J (2013). The emerging spectrum of allelic variation in schizophrenia: current evidence and strategies for the identification and functional characterization of common and rare variants. Mol. Psychiatry.

[3] Purcell SM (2009). Common polygenic variation contributes to risk of schizophrenia and bipolar disorder. Nature.

[4] Hjorthoj C, Sturup AE, McGrath JJ, Nordentoft M (2017). Years of potential life lost and life expectancy in schizophrenia: a systematic review and meta-analysis. Lancet Psychiatry.

[5] Zhou Y, Fan L, Qiu C, Jiang T (2015). Prefrontal cortex and the dysconnectivity hypothesis of schizophrenia. Neurosci. Bull..

[6] Heckers S (2001). Neuroimaging studies of the hippocampus in schizophrenia. Hippocampus.

[7] Sun Y, Collinson SL, Suckling J, Sim K (2019). Dynamic reorganization of functional connectivity reveals abnormal temporal efficiency in schizophrenia. Schizophr. Bull..

[8] Tian Y, Zalesky A, Bousman C, Everall I, Pantelis C (2019). Insula functional connectivity in schizophrenia: subregions, gradients, and symptoms. Biol. Psychiatry Cogn. Neurosci. Neuroimaging.

[9] Giraldo-Chica M, Rogers BP, Damon SM, Landman BA, Woodward ND (2018). Prefrontal-thalamic anatomical connectivity and executive cognitive function in schizophrenia. Biol. Psychiatry.

[10] Dong D, Wang Y, Chang X, Luo C, Yao D (2018). Dysfunction of large-scale brain networks in schizophrenia: a meta-analysis of resting-state functional connectivity. Schizophr. Bull..

[11] Nekovarova T, Fajnerova I, Horacek J, Spaniel F (2014). Bridging disparate symptoms of schizophrenia: a triple network dysfunction theory. Front. Behav. Neurosci..

[12] Venkataraman A, Whitford TJ, Westin CF, Golland P, Kubicki M (2012). Whole brain resting state functional connectivity abnormalities in schizophrenia. Schizophr. Res..

[13] Wu L, Caprihan A, Bustillo J, Mayer A, Calhoun V (2018). An approach to directly link ICA and seed-based functional connectivity: application to schizophrenia. Neuroimage.

[14] Fox MD (2005). The human brain is intrinsically organized into dynamic, anticorrelated functional networks. Proc. Natl. Acad. Sci. USA.

[15] Marrelec G, Fransson P (2011). Assessing the influence of different ROI selection strategies on functional connectivity analyses of fMRI data acquired during steady-state conditions. PLoS One.

[16] Rodionov R (2009). Evaluation of atlas-based segmentation of hippocampi in healthy humans. Magn. Reson Imaging.

[17] Du Y (2015). A group ICA based framework for evaluating resting fMRI markers when disease categories are unclear: application to schizophrenia, bipolar, and schizoaffective disorders. Neuroimage.

[18] Zuo XN (2010). Reliable intrinsic connectivity networks: test-retest evaluation using ICA and dual regression approach. Neuroimage.

[19] Lohmann G (2010). Eigenvector centrality mapping for analyzing connectivity patterns in fMRI data of the human brain. PLoS One.

[20] Zuo XN (2012). Network centrality in the human functional connectome. Cereb Cortex.

[21] Wheeler AL (2015). Further neuroimaging evidence for the deficit subtype of schizophrenia: a cortical connectomics analysis. JAMA Psychiatry.

[22] Skatun KC (2016). Global brain connectivity alterations in patients with schizophrenia and bipolar spectrum disorders. J. Psychiatry Neurosci..

[23] Wang J (2009). Parcellation-dependent small-world brain functional networks: a resting-state fMRI study. Hum. Brain Mapp.

[24] Hayasaka S, Laurienti PJ (2010). Comparison of characteristics between region-and voxel-based network analyses in resting-state fMRI data. Neuroimage.

[25] Hilker R (2018). Heritability of schizophrenia and schizophrenia spectrum based on the nationwide Danish twin register. Biol. Psychiatry.

[26] Trubetskoy V (2022). Mapping genomic loci implicates genes and synaptic biology in schizophrenia. Nature.

[27] Schizophrenia Working Group of the Psychiatric Genomics, C. (2014). Biological insights from 108 schizophrenia-associated genetic loci. Nature.

[28] Elliott LT (2018). Genome-wide association studies of brain imaging phenotypes in UK Biobank. Nature.

[29] Fornito A (2011). Genetic influences on cost-efficient organization of human cortical functional networks. J. Neurosci..

[30] van den Heuvel MP (2013). Genetic control of functional brain network efficiency in children. Eur. Neuropsychopharmacol..

[31] Arnatkeviciute A, Markello RD, Fulcher BD, Misic B, Fornito A (2023). Toward best practices for imaging transcriptomics of the human brain. Biol. Psychiatry.

[32] Xu Z (2022). Meta-connectomic analysis maps consistent, reproducible, and transcriptionally relevant functional connectome hubs in the human brain. Commun. Biol..

[33] Lariviere S (2022). Structural network alterations in focal and generalized epilepsy assessed in a worldwide ENIGMA study follow axes of epilepsy risk gene expression. Nat. Commun..

[34] Xue K (2023). Transcriptional signatures of the cortical morphometric similarity network gradient in first-episode, treatment-naive major depressive disorder. Neuropsychopharmacology.

[35] Keo A (2020). Transcriptomic signatures of brain regional vulnerability to Parkinson’s disease. Commun. Biol..

[36] Li J (2021). Cortical structural differences in major depressive disorder correlate with cell type-specific transcriptional signatures. Nat. Commun..

[37] Estevez-Fraga C (2023). Genetic topography and cortical cell loss in Huntington’s disease link development and neurodegeneration. Brain.

[38] Xue K (2022). Local dynamic spontaneous brain activity changes in first-episode, treatment-naive patients with major depressive disorder and their associated gene expression profiles. Psychol. Med..

[39] Hawrylycz MJ (2012). An anatomically comprehensive atlas of the adult human brain transcriptome. Nature.

[40] Wang L (2016). SchizConnect: mediating neuroimaging databases on schizophrenia and related disorders for large-scale integration. Neuroimage.

[41] Landis D (2016). COINS data exchange: an open platform for compiling, curating, and disseminating neuroimaging data. Neuroimage.

[42] Wang L (2013). Northwestern University Schizophrenia Data and Software Tool (NUSDAST). Front. Neuroinform..

[43] Van Dijk KR, Sabuncu MR, Buckner RL (2012). The influence of head motion on intrinsic functional connectivity MRI. Neuroimage.

[44] Pruim RHR (2015). ICA-AROMA: a robust ICA-based strategy for removing motion artifacts from fMRI data. Neuroimage.

[45] Parkes L, Fulcher B, Yucel M, Fornito A (2018). An evaluation of the efficacy, reliability, and sensitivity of motion correction strategies for resting-state functional MRI. Neuroimage.

[46] Ashburner J (2007). A fast diffeomorphic image registration algorithm. Neuroimage.

[47] Wink AM, de Munck JC, van der Werf YD, van den Heuvel OA, Barkhof F (2012). Fast eigenvector centrality mapping of voxel-wise connectivity in functional magnetic resonance imaging: implementation, validation, and interpretation. Brain Connect..

[48] Fortin JP (2017). Harmonization of multi-site diffusion tensor imaging data. Neuroimage.

[49] Yan CG, Wang XD, Zuo XN, Zang YF (2016). DPABI: data processing & analysis for (resting-state) brain imaging. Neuroinformatics.

[50] Winkler AM, Ridgway GR, Douaud G, Nichols TE, Smith SM (2016). Faster permutation inference in brain imaging. Neuroimage.

[51] Romme IA, de Reus MA, Ophoff RA, Kahn RS, van den Heuvel MP (2017). Connectome disconnectivity and cortical gene expression in patients with schizophrenia. Biol. Psychiatry.

[52] Markello, R. D. et al. Standardizing workflows in imaging transcriptomics with the Abagen toolbox. *Elife* **10**, e72129 (2021). 10.7554/eLife.72129 PMC8660024 34783653

[53] Arnatkeviciute A, Fulcher BD, Fornito A (2019). A practical guide to linking brain-wide gene expression and neuroimaging data. Neuroimage.

[54] Hawrylycz M (2015). Canonical genetic signatures of the adult human brain. Nat. Neurosci..

[55] Chen J, Bardes EE, Aronow BJ, Jegga AG (2009). ToppGene Suite for gene list enrichment analysis and candidate gene prioritization. Nucleic Acids Res..

[56] Szklarczyk D (2019). STRING v11: protein-protein association networks with increased coverage, supporting functional discovery in genome-wide experimental datasets. Nucleic Acids Res..

[57] Keenan AB (2019). ChEA3: transcription factor enrichment analysis by orthogonal omics integration. Nucleic Acids Res..

[58] Park SHE, Kulkarni A, Konopka G (2023). FOXP1 orchestrates neurogenesis in human cortical basal radial glial cells. PLoS Biol..

[59] McClay JL (2011). Genome-wide pharmacogenomic study of neurocognition as an indicator of antipsychotic treatment response in schizophrenia. Neuropsychopharmacology.

[60] Sakamoto M, Hirata H, Ohtsuka T, Bessho Y, Kageyama R (2003). The basic helix-loop-helix genes Hesr1/Hey1 and Hesr2/Hey2 regulate maintenance of neural precursor cells in the brain. J. Biol. Chem..

[61] de Lombares C (2019). Dlx5 and Dlx6 expression in GABAergic neurons controls behavior, metabolism, healthy aging and lifespan. Aging (Albany NY).

[62] Bhaya-Grossman I, Chang EF (2022). Speech computations of the human superior temporal gyrus. Ann. Rev. Psychol..

[63] Friederici AD (2012). The cortical language circuit: from auditory perception to sentence comprehension. Trends Cogn. Sci..

[64] Xu J (2019). Delineating functional segregations of the human middle temporal gyrus with resting‐state functional connectivity and coactivation patterns. Hum. Brain Map.

[65] Vercammen A, Knegtering H, den Boer JA, Liemburg EJ, Aleman A (2010). Auditory hallucinations in schizophrenia are associated with reduced functional connectivity of the temporo-parietal area. Biol. Psychiatry.

[66] Palaniyappan L, Homan P, Alonso-Sanchez MF (2023). Language network dysfunction and formal thought disorder in schizophrenia. Schizophr. Bull..

[67] Patel GH (2021). Failure to engage the temporoparietal junction/posterior superior temporal sulcus predicts impaired naturalistic social cognition in schizophrenia. Brain.

[68] Sherman SM (2016). Thalamus plays a central role in ongoing cortical functioning. Nat. Neurosci..

[69] Guillery RW, Sherman SM (2002). Thalamic relay functions and their role in corticocortical communication. Neuron.

[70] Redinbaugh MJ (2020). Thalamus modulates consciousness via layer-specific control of cortex. Neuron.

[71] Gong J (2019). Evaluation of functional connectivity in subdivisions of the thalamus in schizophrenia. Br. J. Psychiatry.

[72] Woodward ND, Karbasforoushan H, Heckers S (2012). Thalamocortical dysconnectivity in schizophrenia. Am. J. Psychiatry.

[73] Andrews J, Wang L, Csernansky JG, Gado MH, Barch DM (2006). Abnormalities of Thalamic activation and cognition in schizophrenia. Am. J. Psychiatry.

[74] Brandl F (2019). Specific substantial dysconnectivity in schizophrenia: a transdiagnostic multimodal meta-analysis of resting-state functional and structural magnetic resonance imaging studies. Biol. Psychiatry.

[75] Ramet L (2017). Characterization of a human point mutation of VGLUT3 (p.A211V) in the rodent brain suggests a nonuniform distribution of the transporter in synaptic vesicles. J. Neurosci..

[76] McCutcheon RA, Krystal JH, Howes OD (2020). Dopamine and glutamate in schizophrenia: biology, symptoms and treatment. World Psychiatry.

[77] McCutcheon RA, Merritt K, Howes OD (2021). Dopamine and glutamate in individuals at high risk for psychosis: a meta-analysis of in vivo imaging findings and their variability compared to controls. World Psychiatry.

[78] Fremeau RT, Voglmaier S, Seal RP, Edwards RH (2004). VGLUTs define subsets of excitatory neurons and suggest novel roles for glutamate. Trends Neurosci..

[79] Kanno K, Ishiura S (2011). Differential effects of the HESR/HEY transcription factor family on dopamine transporter reporter gene expression via variable number of tandem repeats. J. Neurosci. Res..

[80] Cross-Disorder Group of the Psychiatric Genomics, C. (2013). Identification of risk loci with shared effects on five major psychiatric disorders: a genome-wide analysis. Lancet.

[81] Schizophrenia Psychiatric Genome-Wide Association Study, C. (2011). Genome-wide association study identifies five new schizophrenia loci. Nat. Genet.

[82] Group PGCBDW (2011). Large-scale genome-wide association analysis of bipolar disorder identifies a new susceptibility locus near ODZ4. Nat. Genet.

[83] Hansen JY (2021). Mapping gene transcription and neurocognition across human neocortex. Nat. Hum. Behav..

[84] Ji Y (2021). Genes associated with gray matter volume alterations in schizophrenia. Neuroimage.

[85] Cai M (2024). Homotopic functional connectivity disruptions in schizophrenia and their associated gene expression. Neuroimage.

