# Supplementary material for: Exploring functional dysconnectivity in schizophrenia: alterations in eigenvector centrality mapping and insights into related genes from transcriptional profiles
Source: Schizophrenia (Heidelb). 2024 Mar 15;10(1):37. doi: 10.1038/s41537-024-00457-1 (PMC10943118; doi:10.1038/s41537-024-00457-1)
Supplement: Supplementary file 1 — Supplementary Figure [file 41537_2024_457_MOESM1_ESM.docx]

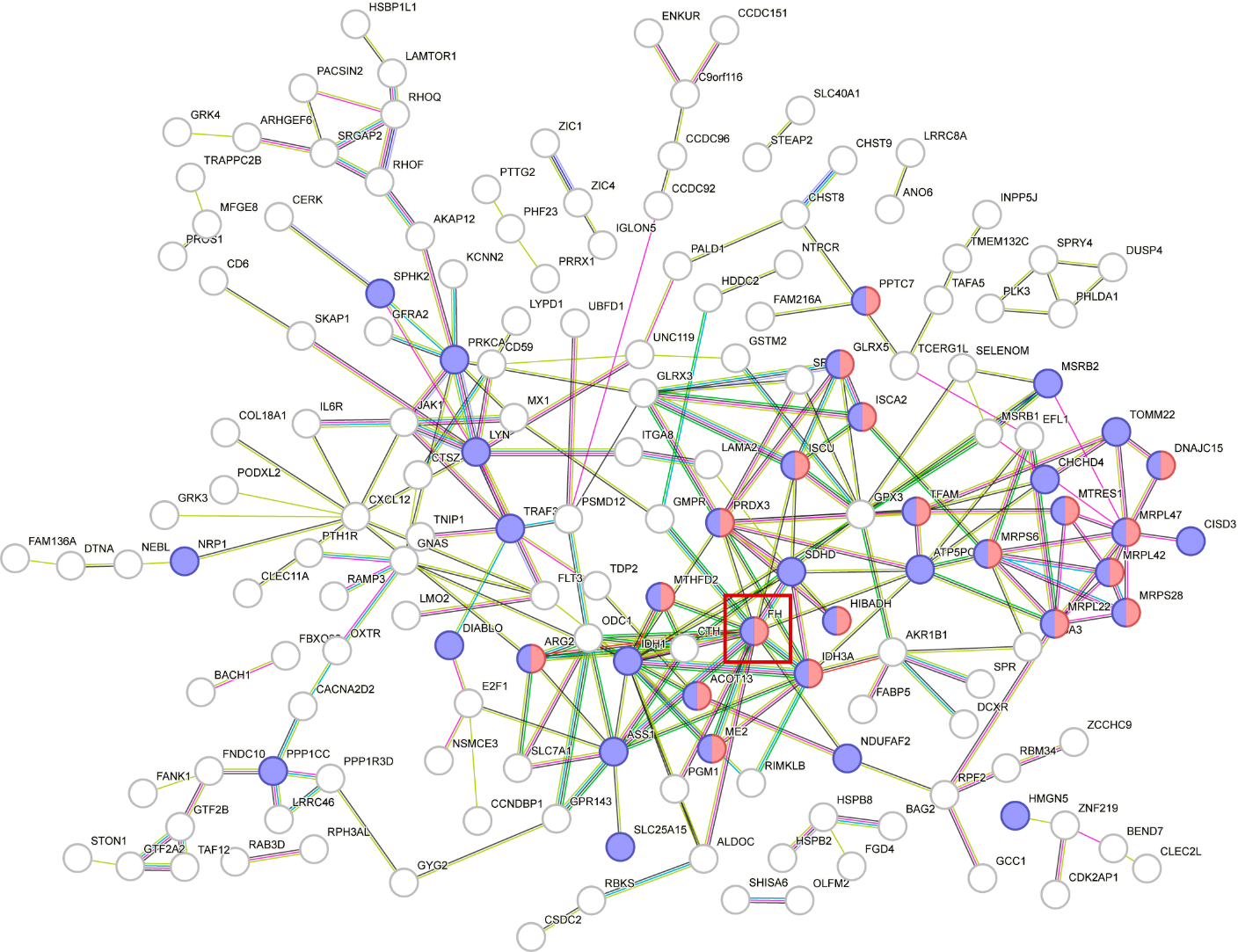
**Supplementary Figure**

**Supplementary Fig. 1 | The PPI network constructed by genes with lower expression in regions with ECM alterations in schizophrenia.** This network consisting of 164 proteins (depicted as spheres) and 248 edges was constructed using 304 genes with higher expression levels in brain regions exhibiting ECM alterations in schizophrenia compared to other gray matter regions. *FH* was identified as one of the central nodes in this network.

Abbreviations: ECM, eigenvector centrality mapping; PPI, protein-protein interaction.
